# Supplementary material for: Third Epidemiological Analysis of Nasopharyngeal Carcinoma in the Central Region of Japan from 2006 to 2015
Source: Cancers (Basel). 2019 Aug 15;11(8):1180. doi: 10.3390/cancers11081180 (PMC6721521; doi:10.3390/cancers11081180)
Supplement: Supplementary file 1 [file cancers-11-01180-s001.pdf]

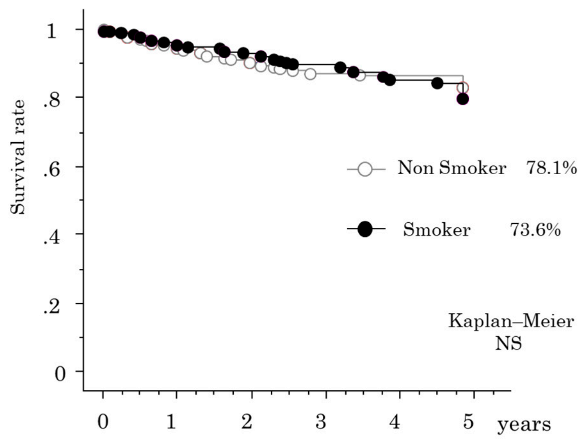

Supplementary Figure S1. Kaplan–Meier curve for overall survival according to smoking history in smokers and nonsmokers.The five-year survival rate was not significantly different from 78.1% for nonsmokers and 73.6% for smokers.

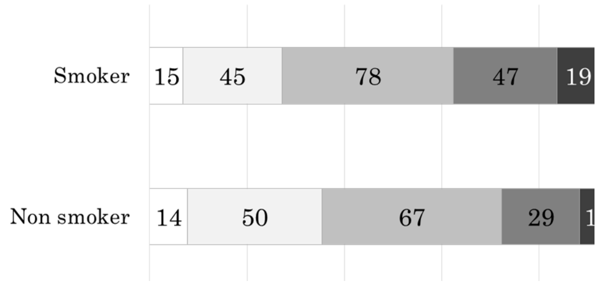

Supplementary Figure S2. Pretherapeutic clinical staging according to smoking history. There was no significant difference based on chi-square test.

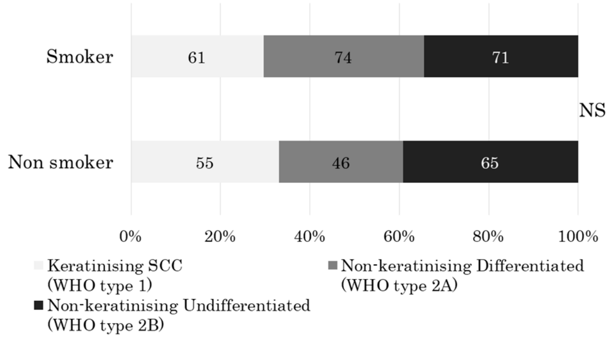

Supplementary Figure S3. Histologically classification according to the 2005 WHO criteria based on smoking history. There was no significant difference based on chi-square test.
